# Clinical characteristics of *Pneumocystis jirovecii* pneumonia in non-HIV patients: a retrospective study

**DOI:** 10.3389/fcimb.2026.1710074

**Published:** 2026-05-28

**Authors:** Mengyuan Li, Junsu Pan, Shuting Ye, Chongyang Wang, Jianhua Luo, Cheng Zheng

**Affiliations:** 1 Department of Pulmonary and Critical Care Medicine, Taizhou Municipal Hospital (Taizhou University Affiliated Municipal Hospital), School of Medicine, Taizhou University, Taizhou, China; 2 Department of Critical Care Medicine, Taizhou Municipal Hospital (Taizhou University Affiliated Municipal Hospital), School of Medicine, Taizhou University, Taizhou, China; 3 Taizhou Key Laboratory of Infection and Tumor Immunology, Taizhou Municipal Hospital, Taizhou, China

**Keywords:** clinical characteristics, HIV, next-generation sequencing, *Pneumocystis jirovecii* pneumonia, severity

## Abstract

**Objective:**

To investigate the clinical characteristics and identify risk factors for severe *Pneumocystis jirovecii* pneumonia (PJP) in human immunodeficiency virus (HIV) -negative patients.

**Methods:**

This retrospective study included 39 non-HIV PJP patients diagnosed by next-generation sequencing (NGS) of bronchoalveolar lavage fluid (BALF) at Taizhou Municipal Hospital between June 2019 and March 2025. Patients were divided into severe (n=18) and non-severe (n=21) groups based on disease severity. Continuous variables were analyzed using Student’s t-test or Mann-Whitney U test, while categorical variables were compared with Fisher’s exact test. Variables showing statistical significance (P<0.05) were subsequently included in multivariable logistic regression analysis to identify differences in clinical characteristics between the two groups.

**Results:**

The mean age of the entire cohort was 62.59 ± 12.59 years, and 64.10% (25/39) were male. The severe group had significantly higher case-fatality rate than the non-severe group (61.1% vs. 0%, *P* < 0.001). Serum procalcitonin (PCT) [0.285 (0.113, 0.643) vs. 0.070 (0.045, 0.110) ng/mL; *P* = 0.003] and lactate dehydrogenase (LDH) [554.00 (465.25, 702.00) vs. 269.00 (246.50, 378.00) IU/L; *P* < 0.001] levels were significantly elevated in the severe group compared with the non-severe group. BALF-based NGS detected co-infections in 76.92% (30/39) of cases, predominantly bacteria (35.90%, 14/39) and Cytomegalovirus (CMV) (30.77%, 12/39) infections. The severe group showed significantly higher CMV co-infection rates than the non-severe group (55.56% vs. 9.52%, *P* = 0.006). Multivariable logistic regression analysis revealed that elevated LDH levels (OR = 1.863, 95% CI 1.153-3.009; *P* = 0.011) and CMV co-infection (OR = 11.477, 95% CI 1.186-111.109; *P* = 0.035) were independent risk factors for severe PJP.

**Conclusions:**

*Pneumocystis jirovecii* pneumonia in non-HIV demonstrated a substantial case-fatality rate. BALF-based NGS may facilitate the detection of co-infections. Elevated LDH levels and CMV co-infection emerged as independent predictors of progression to severe PJP.

## 1 Introduction

*Pneumocystis jirovecii* pneumonia (PJP) is a major contributor to life-threatening opportunistic infections among immunocompromised patients. Previous studies have focused on human immunodeficiency virus (HIV)-infected populations, in whom clinical characteristics and treatment strategies have been well defined (Shibata and Kikuchi, 2019; Wang et al., 2025). However, the widespread use of chemotherapy for malignancies, solid organ transplantation (SOT), and immunosuppressive therapies has led to a rising incidence of non-HIV-associated PJP. Recent estimates indicate that non-HIV-associated PJP cases have surpassed 60% of the global total (Rhoads et al., 2024). Compared with HIV-associated PJP, the non-HIV form is frequently characterized by a more acute onset, more severe hypoxemia, and higher rates of critical illness and in-hospital case-fatality rate, posing a formidable challenge in clinical management (Roux et al., 2014; Gaborit et al., 2019; Wahyuningsih et al., 2021; Kamel et al., 2024). Furthermore, the etiological diagnosis of PJP faces multiple challenges. On the one hand, the colonization rate of *Pneumocystis jirovecii* in the respiratory tract can be as high as 40% (Giacobbe et al., 2023), while the incidence of co-infections may reach 80% (Jiang et al., 2021; Huang et al., 2024). On the other hand, conventional detection methods such as microscopy exhibit low sensitivity (Apostolopoulou and Fishman, 2022), making it difficult to distinguish colonization from infection and often leading to missed detection of co-pathogens. Bronchoalveolar lavage fluid (BALF)-based next-generation sequencing (NGS) enables the simultaneous detection of PJP and a wide range of co-pathogens in a single run, providing rapid and comprehensive pathogen identification for critically ill patients (Society of Clinical Microbiology of China International Exchange and Promotion Association for Medical and Healthcare, 2024; Chinese Thoracic Society, 2023). Currently, studies on risk factors for severe non-HIV PJP are limited. This study aimed to identify risk factors for severe disease and to evaluate the utility of BALF-based NGS for detecting co-infections in non-HIV PJP patients.

## 2 Patients and methods

### 2.1 Study design and patients

**Figure 1:**
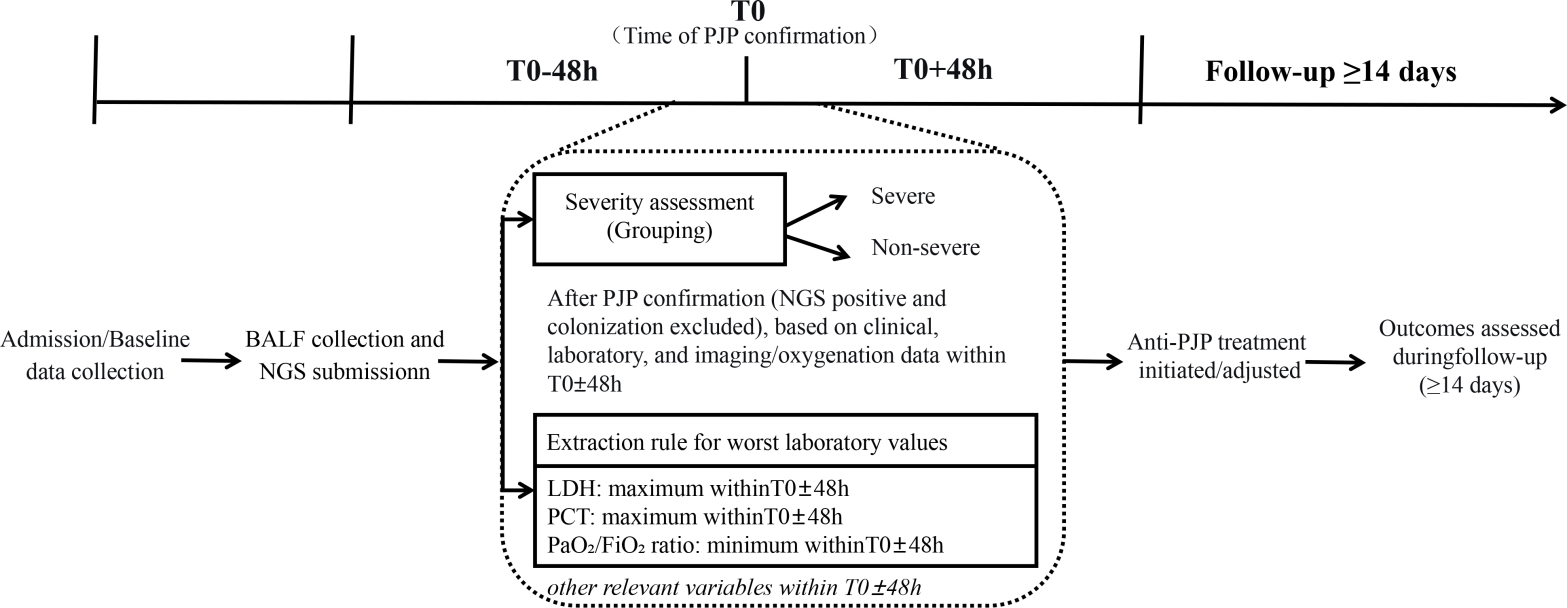
Study timeline of patient enrollment, data collection, severity grouping, and outcome assessment. BALF, bronchoalveolar lavage fluid; LDH, lactate dehydrogenase; NGS, next-generation sequencing; PaO_2_/FiO_2_, partial pressure of arterial oxygen to fraction of inspired oxygen; PCT, procalcitonin; PJP, *Pneumocystis jirovecii* pneumonia.

This retrospective cohort study included 39 consecutive non-HIV-associated PJP patients with confirmed diagnosis and complete clinical data, who were identified from the electronic medical record system of Taizhou Municipal Hospital (Taizhou University Affiliated Municipal Hospital), School of Medicine, between June 2019 and March 2025. A study timeline illustrating patient identification, grouping, data collection, and outcome assessment is provided (**Figure 1**). The inclusion criteria were as follows: (1) presence of pneumonia-related symptoms such as cough, fever, or dyspnea; (2) chest CT showing new ground-glass opacities, consolidation, or patchy exudative lesions; (3) detection of *Pneumocystis jirovecii* nucleic acid sequences via bronchoalveolar lavage fluid (BALF)-based mNGS or tNGS, with results interpreted by at least two experienced attending physicians according to established criteria (Liu et al., 2024) to exclude colonization; (4) receipt of standard anti-PJP treatment and completion of at least 14 days of follow-up. Exclusion criteria included: (1) positive HIV antibody status; (2) absence of microbiological confirmation for PJP; (3) incomplete clinical documentation. The diagnosis of PJP was independently confirmed by two or more chief physicians within 48 hours before and after PJP confirmation, integrating clinical, laboratory, imaging, and treatment response data. This study was approved by the Ethics Committee of Taizhou Municipal Hospital (Taizhou University Affiliated Municipal Hospital), School of Medicine (Ethics Approval No.: LWYJ2025265).

### 2.2 Data collection

The following data were extracted from medical records: (1) basic patient information, (2) immune status, (3) clinical characteristics at PJP onset, (4) the worst laboratory values within the 48-hour window before and after PJP confirmation, (5) the imaging features from the radiological examination closest to the time of PJP confirmation, (6) pathogenic spectra identified by BALF-based NGS, (7) therapeutic strategies, and (8) treatment outcomes assessed after at least 14 days of follow-up.

### 2.3 Definitions

Severe PJP was defined according to the Chinese guidelines for community-acquired pneumonia (CAP) and hospital-acquired pneumonia (HAP) (Qu and Cao, 2016; Shi et al., 2019). Severe CAP was defined as meeting at least one major criterion (invasive mechanical ventilation or septic shock requiring vasopressors) or at least three minor criteria: PaO_2_/FiO_2_ ratio ≤250, multilobar infiltration, confusion, blood urea nitrogen ≥7.14 mmol/L, respiratory rate ≥30/min, or systolic blood pressure <90 mmHg. Severe HAP was defined as requiring invasive mechanical ventilation or septic shock with vasopressor support.

Adjunctive corticosteroid therapy for PJP was defined as corticosteroids initiated or significantly increased (≥50% increase in daily dose) after PJP diagnosis specifically for the management of PJP-related respiratory failure or inflammation. Corticosteroids used solely for the treatment of underlying diseases without dose escalation were not counted as adjunctive therapy.

### 2.4 Bronchoalveolar lavage and microbiological confirmation

All patients underwent thorough pre-procedural evaluation and provided written informed consent for bronchoscopy. The bronchoalveolar lavage site was selected based on chest CT findings: for focal lesions, the lung segment with the most significant radiological abnormalities was chosen; for diffuse lesions, the right middle lobe was lavaged. Three to five aliquots of 25–50 mL sterile saline was instilled and aspirated per segment as needed. BALF samples for mNGS and tNGS were sent to BGI Genomics and ADICON Clinical Laboratories, respectively.

### 2.5 Statistical analysis

All analyses were performed using SPSS software (Version 29.0). All graphical representations were generated using GraphPad Prism 10.0. Categorical data were expressed as numbers (percentage) and were compared using Fisher’s exact test. Normally distributed continuous data were presented as mean ± standard deviation (mean ± SD) and were compared using the independent samples t-test. Non-normally distributed measurement data were expressed as median and interquartile range (IQR), and intergroup comparisons were performed using the Mann-Whitney U test. Variables in the univariate analysis with *P* < 0.05 were considered candidates for building multivariate stepwise logistic regression models. The discriminatory ability of LDH was evaluated by receiver operating characteristic (ROC) curve analysis, and the area under the curve (AUC) was calculated. All statistical tests were two-sided, and a *P*-value < 0.05 was considered statistically significant.

## 3 Results

### 3.1 Clinical characteristics

**Figure 2:**
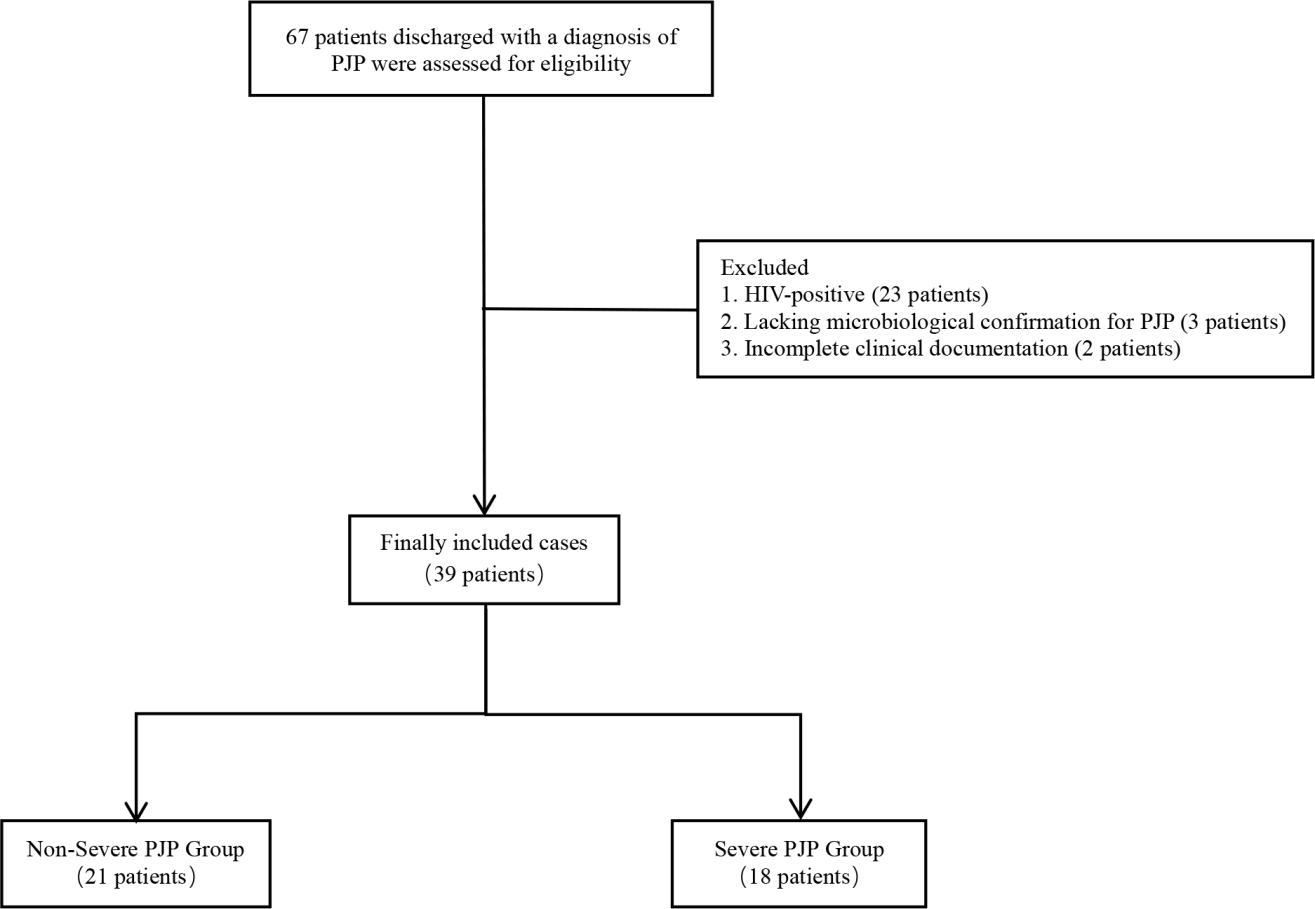
The patient flowchart with respect to inclusion and exclusion. PJP, *Pneumocystis jirovecii* pneumonia; HIV, human immunodeficiency virus.

**Figure 2** shows the patient screening and enrollment process. A total of 39 patients with PJP were included in this study. Among them, 18 (46.15%) were classified as severe cases and 21 (53.85%) as non-severe. The cohort consisted of 25 males (64.10%) and 14 females (35.90%), with a mean age of 62.59 ± 12.59 years and a mean body mass index (BMI) of 23.83 ± 5.46 kg/m². The median duration of hospitalization was 16.00 days (9.00, 20.00). Comorbidities included type 2 diabetes (15/39, 38.46%), primary hypertension (14/39, 35.90%), hematological malignancies (11/39, 28.21%), connective tissue diseases (8/39, 20.51%), solid malignancies (7/39, 17.95%), chronic pulmonary diseases (7/39, 17.95%; three bronchiectasis, four chronic obstructive pulmonary diseases (COPD)), cardiovascular diseases (5/39, 12.82%), and neurological disorders (4/39, 10.26%). Additionally, eight patients (20.51%) had two or more comorbidities. Regarding immunosuppressive therapy, 18 patients (46.15%) had received glucocorticoids prior to admission. Among these, the median daily prednisolone-equivalent dose was 30.00 (18.75, 41.25) mg, and nine patients (23.08%) had been on glucocorticoids for more than three months. In addition, 11 (28.21%) were on other immunosuppressants, including cyclophosphamide (n=4), tacrolimus (n=2), rituximab (n=2), methotrexate (n=1), ciclosporin (n=1), tocilizumab (n=1), and bendamustine (n=1). Furthermore, 13 (33.33%) patients had undergone chemotherapy or thoracic radiotherapy within the preceding 6 months. Only two patients (5.1%) had received PJP prophylaxis prior to diagnosis, both in the severe group. The most frequent clinical symptoms were fever (30/39, 76.92%), cough (35/39, 89.74%), and dyspnea (33/39, 84.62%). Imaging features indicated bilateral ground-glass opacities in all patients, accompanied by consolidation (26/39, 66.67%), pleural effusion (9/39, 23.08%), cavitation (3/39, 7.69%), and pneumothorax (2/39, 5.13%). No statistically significant differences were observed between the severe and non-severe groups in terms of general characteristics, comorbidities, immunosuppressive status, clinical manifestations, or imaging features (**Table 1**).

**Table 1:** Clinical and imaging features of 39 patients with non-HIV PJP.

Variable	Total (n=39)	Non-severe PJP group (n=21)	Severe PJP group (n=18)	t/Z	*P* value	
Age, years,( ± s)	62.59 ± 12.59	66 ± 12.26	58.61 ± 12.11	1.887	0.067
male, n (%)	25 (64.10)	15 (71.43)	10 (55.56)	-	0.337
BMI, Kg/m^2^, ( ± s)	23.83 ± 5.46	24.74 ± 6.36	22.37 ± 3.27	1.43	0.163
Onset to diagnosis, days, median (IQR)	11.00 (7.00, 17.00)	11.00 (6.00, 15.50)	11.00 (7.75, 17.25)	-0.212	0.832
Admission to diagnosis, days, median (IQR)	7.00 (4.00, 11.00)	6.00 (4.50, 10.00)	7.00 (3.00, 13.75)	0	>0.999
Duration of hospitalization, days, median (IQR)	16.00 (9.00, 20.00)	13.00 (8.00, 20.00)	17.00 (9.00, 21.75)	-0.466	0.641
Comorbidity, n (%)						
Type 2 diabetes	15 (38.46)	8 (38.09)	7 (38.89)	-	>0.999
Primary hypertension	14 (35.90)	10 (47.62)	4 (22.22)	-	0.180
Hematologic malignancy	11 (28.21)	6 (28.57)	5 (27.78)	-	>0.999
Connective tissue disease	8 (20.51)	3 (14.29)	5 (27.78)	-	0.432
Chronic pulmonary disease	7 (17.95)	5 (23.81)	2 (11.11)	-	0.418
Solid malignancy	7 (17.95)	4 (19.05)	3 (16.67)	-	>0.999
Cardiovascular disease	5 (12.82)	2 (9.52)	3 (16.67)	-	0.647
Cerebrovascular disease	4 (10.26)	1 (4.76)	3 (16.67)	-	0.318
Immunosuppressive status, n (%)						
Glucocorticoids	18 (46.15)	8 (38.10)	10 (55.56)	-	0.343
Prednisolone-equivalent dosage, mg/day, median (IQR)	30.00 (18.75, 41.25)	30.00 (16.25, 40.00)	32.50 (18.75, 50.00)	-0.806	0.420
Glucocorticoid duration >3 months, n (%)	9 (23.08)	4 (19.05)	5 (27.78)	-	0.637
Other immunosuppressants	11 (28.21)	5 (23.81)	6 (33.33)	-	0.723
Undergoing chemotherapy or chest radiotherapy within 6 months	13 (33.33)	6 (28.57)	7 (38.89)	-	0.520
Symptoms, n (%)						
Fever	30 (76.92)	16 (76.19)	14 (77.78)	-	>0.999
Dyspnea	33 (84.62)	17 (80.95)	16 (88.89)	-	0.667
Cough	35 (89.74)	19 (90.48)	16 (88.89)	-	>0.999
Imaging features, n (%)					
GGO	39 (100.00)	28 (100.00)	11 (100.00)	-	-
consolidation	26 (66.67)	13 (61.90)	13 (72.22)	-	0.734
Pleural effusion	9 (23.08)	2 (9.52)	7 (38.89)	-	0.055
Cavitation	3 (7.69)	2 (9.52)	1 (5.56)	-	>0.999

HIV, human immunodeficiency virus; PJP, *Pneumocystis jirovecii* pneumonia; BMI, body mass index; IQR, interquartile range; GGO, ground-glass opacity.

### 3.2 Laboratory findings

The laboratory parameters of the two groups (with the worst values extracted within the 48-hour window before and after PJP confirmation) are summarized in **Table 2**. In comparison with the non-severe group, the severe group exhibited markedly higher levels of serum PCT [0.285 (0.113, 0.643) vs. 0.070 (0.045, 0.110) ng/mL; *P* = 0.003] and LDH [554.00 (465.25, 702.00) vs. 269.00 (246.50, 378.00) IU/L; *P* < 0.001], while a markedly lower PaO_2_/FiO_2_ [118.00 (70.00, 202.00) vs. 264.00 (212.25, 368.00) mmHg; *P* < 0.001]. No statistically significant differences were observed in the remaining laboratory parameters between the two groups.

**Table 2:** Laboratory findings of 39 patients with non-HIV PJP.

Laboratory findings	Total (n=39)	Non-severe PJP group (n=21)	Severe PJP group (n=18)	Z value	*P* value
White blood cell count, median (IQR), ×10^9^/L	9.40 (4.90, 13.50)	7.90 (4.40, 13.80)	9.90 (4.95, 12.80)	-0.507	0.612
Neutrophil count, median (IQR), ×10^9^/L	7.49 (4.11, 12.39)	6.46 (2.93, 12.37)	8.60 (6.34, 12.44)	-1.380	0.167
Lymphocyte count, median (IQR), ×10^9^/L	0.85 (0.35, 1.22)	0.87 (0.52, 1.41)	0.71 (0.29, 1.09)	-1.014	0.310
Platelet count, median (IQR), ×10^9^/L	166.00 (124.00, 258.00)	165 (134.00, 267.50)	184.00 (64.75, 255.00)	-0.578	0.564
hsCRP, median (IQR), mg/L	71.33 (30.91, 93.60)	65.77 (21.65, 92.90)	77.00 (39.94, 104.16)	-0.901	0.367
PCT, median (IQR), ng/ml	0.110 (0.060, 0.390)	0.070 (0.045, 0.110)	0.285 (0.113, 0.643)	-2.977	0.003
Lactate, median (IQR), mmol/l	1.75 (1.30, 2.43)	1.60 (1.23, 2.28)	2.00 (1.38,2.58)	-1.156	0.248
Oxygenation index, median (IQR), mmHg	226.00 (145.00,300.00)	264.00 (212.25,368.00)	118.00 (70.00, 202.00)	-4.14	<0.001
Total bilirubin, median (IQR), μmol/L	8.20 (6.10, 13.60)	9.40 (6.55, 14.35)	8.15 (5.88, 12.85)	-0.338	0.735
urea nitrogen, median (IQR), μmol/L	5.10 (3.50, 8.70)	4.10 (3.30, 6.80)	5.65 (3.80, 10.00)	-1.395	0.163
Creatinine, median (IQR), μmol/L	62.00 (47.60, 96.00)	61.70 (52.50, 95.50)	62.50 (44.13, 96.25)	-0.423	0.673
LDH, median (IQR), IU/L	390.00 (268.00, 566.00)	269.00 (246.50, 378.00)	554.00 (465.25, 702.00)	-4.099	<0.001
ALP, median (IQR), IU/L	84.00 (62.50, 111.25)	79.50 (52.75, 99.00)	88.50 (64.75, 116.50)	-0.994	0.320
ALT, median (IQR), IU/L	25.00 (15.00, 45.00)	25.00 (15.50, 30.50)	26.00 (14.50, 81.75)	-0.790	0.430
Serum albumin, median (IQR), g/L	30.70 (27.70, 35.90)	33.60 (28.75, 37.00)	29.55 (26.60, 34.88)	-1.649	0.099
Serum (1,3)-β-D-glucan test, n (%)	31 (79.49)	15 (78.95)	16 (88.89)	-	0.660

HIV, human immunodeficiency virus; PJP, *Pneumocystis jirovecii* pneumonia; hsCRP, high-sensitivity C-reactive protein; PCT, procalcitonin; LDH, lactate dehydrogenase; ALP, alkaline phosphatase; ALT, alanine aminotransferase.

### 3.3 Pathogen detection by BALF-based NGS

**Figure 3:**
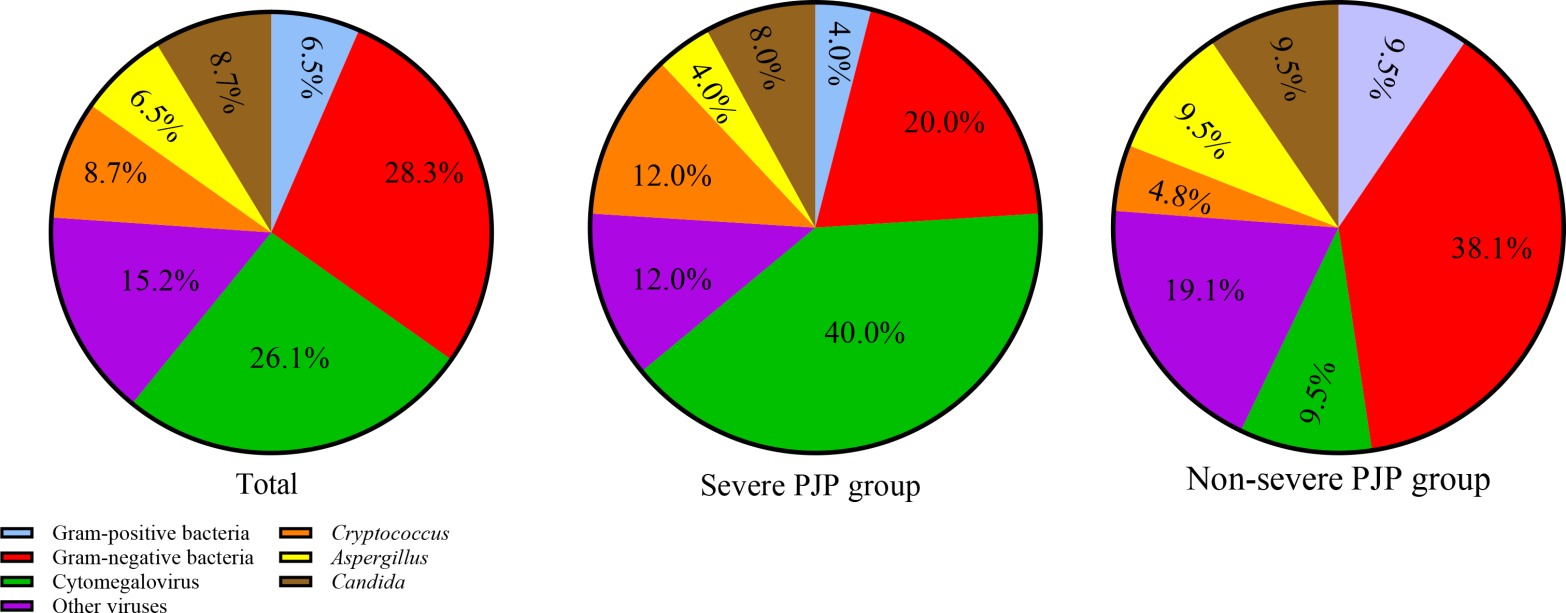
Comparative distribution of detected pathogens among co-infected PJP patients. Pie charts depict the pathogen distribution for all co-infected patients (n=30), the severe PJP group (n=15), and the non-severe PJP group (n=15), based on the total number of detected pathogens (46 total: 25 in the severe group, 21 in the non-severe group). In the severe group, CMV was the most frequently detected pathogen; in the non-severe group, gram-negative bacteria were the most frequent. PJP, *Pneumocystis jirovecii* pneumonia; CMV, Cytomegalovirus.

All 39 patients with PJP were definitively diagnosed by NGS of BALF, with 27 cases tested using mNGS and 12 using tNGS. Co-infections were identified in 30 patients, 12 of whom harbored two or more pathogens. The distribution of detected pathogens in the two groups is shown in **Figure 3**. The rate of CMV co-infection was significantly higher in the severe group than in the non-severe group (55.56% vs. 9.52%, *P* = 0.006), whereas the distribution of other pathogens did not differ between the two groups.

### 3.4 Analysis of severe risk factors

**Figure 4:**
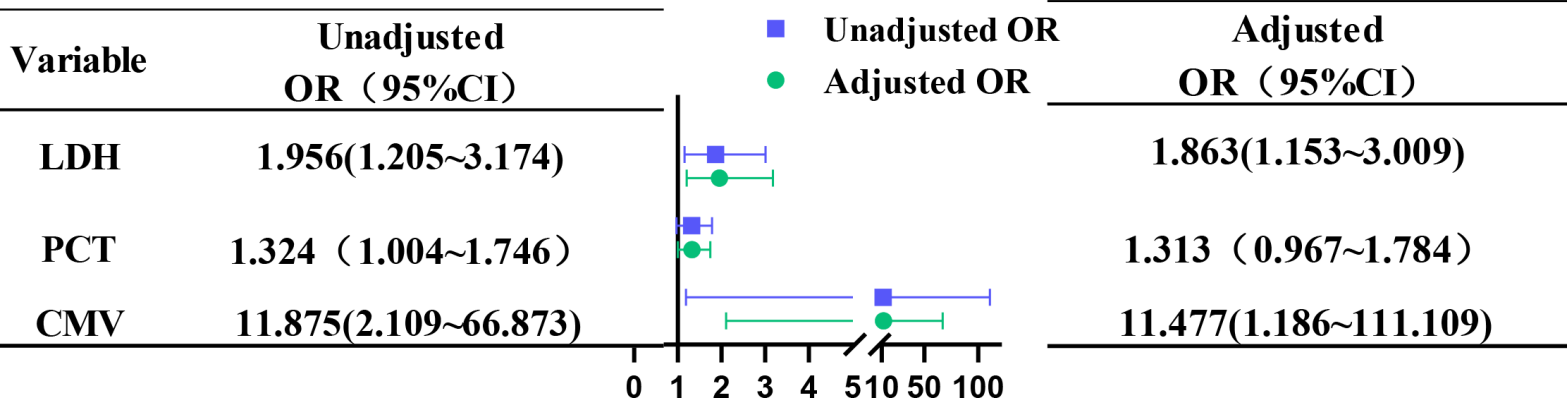
Multivariable logistic regression identifying independent risk factors for severe non-HIV PJP. Forest plot displays significant predictors with adjusted odds ratios (95% CIs): elevated serum LDH and CMV co-infection. HIV, human immunodeficiency virus; PJP, *Pneumocystis jirovecii* pneumonia; CI, confidence interval; OR, odds ratio; LDH, lactate dehydrogenase; PCT, procalcitonin; CMV, Cytomegalovirus.

**Figure 5:**
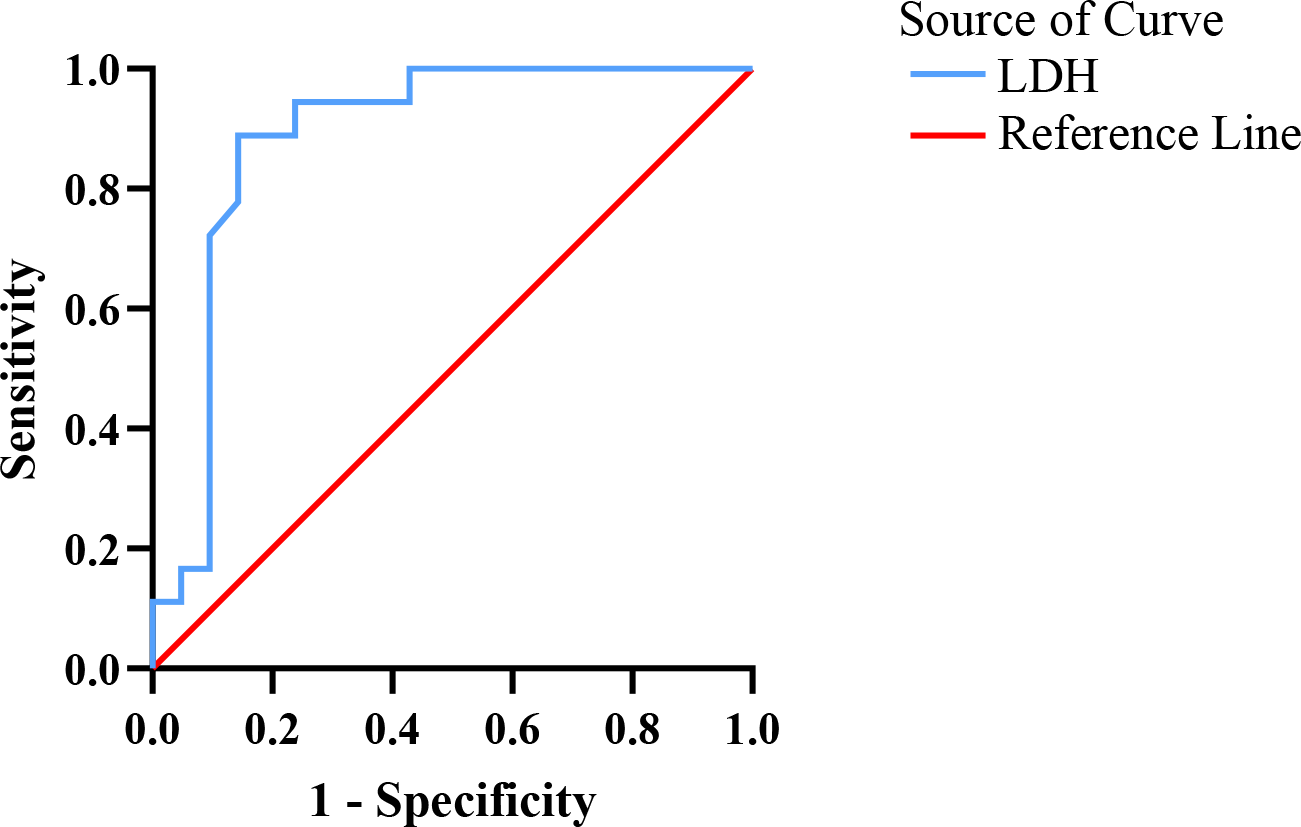
ROC curve of serum LDH for predicting severe PJP. The optimal LDH cut-off value was 399.5 U/L (Youden’s index=0.746), yielding a sensitivity of 88.9% and specificity of 85.7%. The AUC was 0.885 (95% CI: 0.769–1.001, *P* < 0.001). ROC, receiver operating characteristic; LDH, lactate dehydrogenase; PJP, *Pneumocystis jirovecii* pneumonia; AUC, area under the curve.

Univariable logistic regression analysis identified elevated serum LDH (OR = 1.956, 95% CI: 1.205–3.174; *P* = 0.007), elevated PCT (OR = 1.324, 95% CI: 1.004–1.746; *P* = 0.047), and CMV co-infection (OR = 11.875, 95% CI: 2.109–66.873; *P* = 0.005) as risk factors for severe non-HIV PJP. After incorporating these factors into a multivariable logistic regression model, elevated serum LDH (OR = 1.863, 95% CI 1.153-3.009; *P* = 0.011) and CMV co-infection (OR = 11.477, 95% CI: 1.186–111.109; *P* = 0.035) remained independent risk factors for severe non-HIV PJP (**Figure 4**). Furthermore, the predictive performance of LDH was evaluated using ROC curve analysis. The AUC for LDH in predicting severe PJP was 0.885 (95% CI: 0.769–1.000; P < 0.001). The optimal cutoff value was determined to be 399.5 U/L (Youden’s index=0.746), yielding a sensitivity of 88.9% and a specificity of 85.7% (**Figure 5**).

### 3.5 Treatment and outcomes

The initial treatment regimen was trimethoprim-sulfamethoxazole (TMP-SMX) for 38 patients, whereas one patient with a documented sulfonamide allergy received clindamycin plus primaquine. Guided by the NGS findings, the antimicrobial therapy was modified in 24 patients. During the treatment course, two patients with rapid clinical deterioration received adjunctive caspofungin therapy, and one patient was switched to clindamycin plus primaquine due to the development of a severe rash.

Regarding adjunctive corticosteroid therapy for PJP, 77.8% (14/18) of patients in the severe group and 33.3% (7/21) in the non-severe group received adjunctive corticosteroids. The initial daily prednisolone-equivalent dose was [100.00 (50.00, 100.00) vs. 50.00 (30.00, 100.00) mg], the cumulative in-hospital dose was [500.00 (280.00, 525.00) vs. 525.00 (300.00, 1092.50) mg], and the in-hospital treatment duration was [9.50 (3.00, 18.50) vs. 11.00 (10.00, 14.00) days], respectively. The severe group exhibited a significantly higher incidence of complications compared to the non-severe group. Specifically, shock requiring vasopressor support occurred in 33.33% (6/18) of patients in the severe group, and 61.11% (11/18) required respiratory support, including invasive mechanical ventilation in 44.44% (8/18) and non-invasive ventilation in 16.67% (3/18). In contrast, only one patient (4.76%) in the non-severe group required non-invasive ventilation. The overall case-fatality rate was 28.2% (11/39). All fatalities occurred in the severe group, corresponding to a mortality rate of 61.11% (11/18) within this subgroup. Comparative analyses revealed that the severe group had significantly higher rates of shock (33.33% vs. 0%; *P* = 0.006), need for respiratory support (61.11% vs. 4.76%; *P* < 0.001), and mortality (61.11% vs. 0%; *P* < 0.001) than the non-severe group (**Table 3**).

**Table 3:** Clinical outcomes and treatment interventions in patients with non-HIV PJP.

Complication	Total (n=39)	Non-severe PJP group (n=21)	Severe PJP group (n=18)	*P* value
Shock	6	0	6	0.006
ICU Admission	11	1	10	0.001
Mechanical Ventilation	12	1	11	<0.001
Mortality	11	0	11	0.001

HIV, human immunodeficiency virus; PJP, *Pneumocystis jirovecii* pneumonia; ICU, intensive care unit.

## 4 Discussion

*Pneumocystis jirovecii* pneumonia is more likely to progress rapidly to severe disease in non-HIV-infected people compared to HIV-infected people (Gaborit et al., 2019). Severe PJP significantly increases the need for mechanical ventilation and the risk of mortality, with reported case-fatality rates exceeding 65% (Festic et al., 2005; Weng et al., 2016; Wang et al., 2022). In our cohort, 46.15% of patients met the diagnostic criteria for severe pneumonia. In the severe group, the mechanical ventilation rate was as high as 61.11%, and the mortality rate reached 61.11%, further emphasizing the importance of early identification of high-risk patients. Notably, despite the worse outcomes in the severe group, there were no significant differences between the two groups in demographic characteristics (age, sex, BMI), comorbidities, immunosuppressive status, clinical symptoms, or imaging features. However, the severe group exhibited significantly higher serum LDH and PCT levels and a higher rate of CMV co-infection. Multivariate analysis revealed that elevated serum LDH levels and co-infection with CMV are important features and independent risk factors for progression to severe disease in non-HIV PJP patients.

It is well established that major risk factors for non-HIV PJP include underlying immunosuppressive conditions (e.g., malignancies, transplantation) and immunosuppressive agents (e.g., glucocorticoids, chemotherapeutic drugs, monoclonal antibodies) (Lee et al., 2025). However, studies specifically examining whether these factors are associated with PJP severity remain limited. In the present study, we found no significant differences between severe and non-severe groups in terms of underlying diseases, immunosuppressive therapies, or comorbidities. A large multicenter study (Lécuyer et al., 2024) reported that solid malignancies, immune-mediated inflammatory diseases, and long-term corticosteroid use (especially ≥10 mg/day) were independently associated with mortality. Another large study (Chen et al., 2022) of 485 PJP patients in China found that while there were no significant differences in the rates of respiratory failure or non-invasive ventilation across different underlying diseases, significant differences were observed in ICU admission rates, rates of invasive mechanical ventilation, and in-hospital mortality. In contrast, a prospective study (Gaborit et al., 2019) demonstrated that the distribution of underlying immunosuppressive conditions, corticosteroid use, and chronic comorbidities did not differ significantly between severe and non-severe PJP groups. However, existing evidence remains divergent, suggesting that factors influencing PJP severity may vary across populations, multicenter cohorts.

*Pneumocystis jirovecii* infection damages alveolar epithelial, leading to disruption of the alveolar-capillary barrier and diffuse alveolar injury (Thomas and Limper, 2007). This damage leads to the release of LDH from injured lung tissue into the bloodstream, resulting in elevated serum LDH levels (Smith et al., 1988). Serum LDH level can be considered a biochemical indicator of the extent of lung injury, with elevation often indicating more severe hypoxemia (Tasaka et al., 2007). In non-HIV patients with PJP, serum LDH levels are closely associated with disease severity and final prognosis (Koh et al., 2024; Sun et al., 2021). In this study, we found that LDH was an independent risk factor for progression of the disease to a critically ill condition (OR = 1.863, 95% CI 1.153-3.009; *P* = 0.011). Our result was consistent with that of a prior study (Gaborit et al., 2019) which together supported that LDH levels were positively correlated with disease severity. A subsequent study (Koh et al., 2024) further expanded the value of the clinical application of LDH, as their construction of a multivariate risk scoring model was able to effectively predict the risk of death in non-HIV PJP patients, and the results showed that the LDH levels of non-survivors were close to twice those of survivor patients. In addition, LDH can also be used as an auxiliary diagnostic tool for PJP, while most previous studies have focused on HIV-infected patients (Esteves et al., 2014, 2015; Deng et al., 2018), with fewer relevant reports in non-HIV-infected patients. One study (Sun et al., 2021) firstly explored the value of LDH as an auxiliary diagnostic tool for non-HIV PJP. The authors found that, using 379 U/L as the cut-off value, the sensitivity for identifying non-HIV PJP amounted to 85% and the specificity was 77%. Our study also yielded similar results. Further analysis using 399.5 U/L as a potential cutoff value revealed that LDH could serve as an early warning indicator for the development of severe disease in PJP, with a sensitivity of 88.9% and a specificity of 85.7%. Based on current evidence, we propose that serum LDH testing has important clinical significance: (1) it can be used as an early warning indicator of disease severity; (2) it aids in prognostic assessment; (3) it provides a reference for therapeutic decision making. Therefore, for suspected or confirmed non-HIV PJP patients, routine dynamic monitoring of LDH may assist clinical assessment.

Procalcitonin, as a sensitive biomarker of infection, has been shown in studies (Chae et al., 2021; Chen et al., 2025) to effectively reflect the severity of infection in immunocompromised patients. However, research on PCT in PJP remains relatively limited. A retrospective study demonstrated that PCT levels may predict poor prognosis in PJP patients with COPD, with univariate regression analysis indicating that each one-unit increase in PCT was associated with a 14.9-fold increase in mortality risk (Feng and Tong, 2024). Notably, even in PJP patients without underlying COPD, PCT level remained an independent risk factor for in-hospital mortality. Furthermore, serum PCT concentrations may aid in differentiating PJP from other pathogen infections (Mendelson et al., 2018); specifically, PCT levels in PJP patients were significantly lower than those in patients with bacterial pneumonia, with a sensitivity of 79% and specificity of 83% for differentiation, although its discriminative value is limited in cases of mixed infection (Mendelson et al., 2018). In the present study, the severe group had significantly higher PCT levels than the non-severe group (0.285 vs. 0.070, *P* = 0.003), which, combined with the higher rate of mixed infection in this group, suggests that elevated PCT may reflect disease progression in PJP and an increased risk of co-infection with other pathogens. In addition, other biomarkers such as C-reactive protein, serum albumin, neutrophil and lymphocyte counts have been shown to correlate with disease severity and poor prognosis in non-HIV PJP patients (Gaborit et al., 2019; Lécuyer et al., 2024; Akahane et al., 2021; Winichakoon et al., 2025). However, studies on laboratory markers to predict PJP severity and prognosis are still limited, and definitive conclusions in this regard need to be validated by more and larger prospective studies.

An important advantage of NGS is its ability to detect co-infections. Previous studies have reported that the co-infection rate in patients with PJP exceeds 80%, with CMV being the most prevalent pathogen (Jiang et al., 2021; Huang et al., 2024). A retrospective analysis (Lee et al., 2020) of 52 non-HIV PJP patients revealed a CMV co-infection rate of 26.9%. Those with co-infection had more severe pneumonia (*P* = 0.002) and a mortality rate twice that of patients with PJP alone. These findings were further supported by a study of 80 post-renal transplantation PJP patients (Zou et al., 2022), which reported a higher CMV co-infection rate of 46.25%, significantly associated with increased mortality, higher ICU admission rates, and delayed radiographic resolution.

In our study, the overall co-infection rate among PJP patients was 76.92%, with a CMV co-infection rate of 30.77%. This aligns with a previous report (Lee et al., 2020) but is lower than rates observed in specific immunocompromised subgroups such as renal transplant recipients (Zou et al., 2022). This variation likely reflects differences in study populations, underlying diseases, and degrees of immunosuppression. Notably, the CMV co-infection rate in the severe group reached 55.56% and was associated with significantly higher mortality, highlighting the role of CMV as an indicator of disease severity and poor prognosis. A recent large retrospective cohort study of 249 PJP patients (Perret et al., 2024) showed that a CMV load ≥3 log_10_ copies/mL in BALF or blood was associated with significantly higher ICU admission rates (BALF: 78.4% vs. 28.4%; blood: 68.7% vs. 29.4%; both *P* < 0.001) and twice the length of ICU stays. Moreover, a gradient increase in ICU admission was observed with each 1-log_10_ increase in CMV load, indicating that CMV is not merely a bystander in immunosuppression but may play an active role in lung injury.

These findings corroborate our NGS-based results and further support the hypothesis that CMV exacerbates PJP. The mechanisms may include: (1) inhibition of antigen-presenting cells and helper T cells, worsening immunosuppression; (2) damage to the alveolar-capillary barrier, as reflected by a higher incidence of severe hypoxemia (PaO_2_/FiO_2_ ≤100 mmHg; *P* = 0.035); and (3) induction of excessive inflammatory responses that impede tissue repair and delay recovery (Zou et al., 2022; Yu et al., 2017; Perret et al., 2024). These findings collectively underscore the clinical value of NGS for early CMV detection. Furthermore, since multiple studies (Zou et al., 2022; Yu et al., 2017; Perret et al., 2024) have indicated that CMV co-infection and high CMV load are strongly linked to disease severity and poor outcomes, antiviral therapy against CMV should be considered for PJP patients in whom CMV co-infection is detected via NGS or PCR. Future prospective studies are needed to establish optimal treatment thresholds and confirm the clinical benefit of anti-CMV strategies.

Our study has several limitations. Firstly, as a single-center retrospective investigation, it is subject to inherent biases associated with its design. Secondly, the small sample size in each group limits the statistical power and may compromise the generalizability of our findings. Thirdly, the diagnosis of PJP relied solely on BALF-based NGS results from a single institution, interpreted by two physicians, and lacked validation through conventional methods such as GMS staining or PCR, which may influence diagnostic accuracy. In the future, larger, multicenter prospective studies are needed and more standardized diagnostic criteria should be established to validate the results of this study. Finally, there is currently no universally accepted severity classification for non-HIV PJP. We adopted the Chinese CAP and HAP guidelines, which is a practical approach but has not been specifically validated for PJP, potentially limiting comparisons across studies.

## 5 Conclusion

*Pneumocystis jirovecii* pneumonia in non-HIV patients is a life-threatening opportunistic infection with substantial mortality. Elevated serum LDH levels and CMV co-infection are important clinical characteristics and independent risk factors for progression to severe disease. Severe PJP leads to serious consequences, including shock, respiratory support requirement, and death. Additionally, BALF-based NGS technology can effectively identify co-infections, providing reliable evidence for clinical diagnosis and treatment. These findings contribute to the early identification of high-risk patients and guide clinical intervention. Further research is needed to optimize diagnostic and therapeutic strategies.

## Data Availability

The datasets presented in this article are not readily available because they contain sensitive/identifiable information from human subjects that cannot be shared in open access repositories for legal/privacy reasons. Anonymized data will be shared upon reasonable request from any qualified investigator. Requests should be directed to the corresponding authors.
